# Mrs. George at Cardiff

**Published:** 1918-11-09

**Authors:** 


### Mrs. George at Cardiff.

PRINCE OF WALES' HOSPITAL FOR LIMBLESS MEN.

An interesting ceremony took place on October 24 at the Prince of Wales' Hospital for Limbless Sailors and Soldiers, Cardiff, when Mrs. Lloyd George, as president of the Council, unveiled in the hospital grounds a mural tablet recording the services to the institution of the "James Howell family," and especially the gift of the Parade Hall and adjacent buildings. The history of the hospital having been briefly recounted by Col. Lynn Thomas, C.B., C.M.G., the officer commanding, Mrs. Lloyd George, amid cheers, drew back "Y Ddraig Goch," which covered the stone. Mr. T. Francis Howell, who was accompanied by his sisters, Mrs. J. T. Edwards and Miss Mabel Howard, in thanking Mrs. Lloyd George for her kind references to the family, suggested that it was a big ceremony for a little gift. The company afterwards inspected the new cromlech, training ground, and workshops, which are now complete. Later Mrs. Lloyd George went to Llandaff North and inspected the Edward Nicholl Home for Waifs and Strays, where there are twenty-one babies. In the afternoon Mrs. Lloyd George presided at a Council meeting of the Prince of Wales' Hospital, when it was announced that the endowment fund had now reached £36,413, which has been invested in National War Bonds. Cheques for 1,000 guineas each for the endowment of beds have been received from Sir William James Thomas and Lady Thomas, Councillor J. C. Gould, who has been elected a member of the Council, Mr. and Mrs. J. T. Duncan, and Mr. C. Keppell. Mr. Sidney H. Byass, J.P., has sent a gift of £1,000 in War Bonds, while £500 has come to hand from an anonymous donor. Mrs. J. P. Cadogan has offered a bust of her late husband, one of the founders of the institution, which the Council has accepted for placing in the hospital.

